# Effect of organic acids on growth performance, intestinal morphology, and immunity of broiler chickens with and without coccidial challenge

**DOI:** 10.1186/s13568-021-01299-1

**Published:** 2021-10-20

**Authors:** Ahsan Mustafa, Shiping Bai, Qiufeng Zeng, Xuemei Ding, Jianping Wang, Yue Xuan, Zhuowei Su, Keying Zhang

**Affiliations:** Institute of Animal Nutrition, Key Laboratory for Animal Disease-Resistance Nutrition of China, Ministry of Education, Sichuan Agricultural University, Chengdu, 611130 People’s Republic of China

**Keywords:** Organic acids, SCFA, Acidifiers, Coccidial challenge, Intestinal characteristics, Microbiota, Broilers

## Abstract

A total of 360-day-old broiler chicks were allocated into six groups in 2 (Coccidial challenge or not) × 3 (dietary treatments) factorial design. Three dietary treatments including: basic diet, basic diet plus organic acids (OAs) in drinking water, and basic diet plus OAs in the feed with and without coccidial challenge. The OAs in water or feed improved (P < 0.01) average body weight (ABW), average body weight gain (ABWG), and feed conversion ratio (FCR) as compared with the control diet during starter, grower, and whole experimental period. Coccidial challenge decreased BW, ABWG, and average feed intake (AFI), as well as resulted in poor FCR during the starter and whole experimental period (P < 0.05). Though there was no interaction between OAs supplementation and coccidial challenge, the OAs supplementation improved the overall performance with and without coccidial challenge birds on 21 d and 35 d. IgG was found higher (P = 0.03) in broilers fed OAs in feed without the coccidial challenge group. On 18 d, OAs supplementation in feed increased TNF-γ (P = 0.006), whereas the coccidial challenge decreases TNF-γ (P = 0.01) and IL-10 (P =  < .0001), and increases IgM (P = 0.03), IgG (P = 0.04) and IgA (P = 0.02). On 29 d, the coccidial challenge increases IgM and IgA. On 18 d, jejunal lesion score was found significantly higher in the coccidial challenged group as compared to OAs supplementation with coccidial challenged groups on 18 d (P < 0.0001) and 29 d (P = 0.03). Crypt depth was higher, and Villus-height to Crypt depth ratio was lower in the coccidial challenge group on 18 and 29 d. The Goblet cells were found higher in the non-coccidial challenge on 18 d. After 18 d, 16S rDNA gene sequence analysis of ileal chyme has shown that coccidial challenge decreases *Lactobacillus_reuteri* species as compared to the non-challenged group (P = 0.02). After 29, *Cyanobacteria* abundance reduced (P = 0.014) in the challenged group than the non-challenged group at the phylum level. At the genus level, *Lactobacillus* (P = 0.036) and *unidentified Cyanobacteria* (P = 0.01) were found higher in the non-challenged group than the coccidial challenge group. The results indicate that the OAs supplementation showed improved responses in a pattern similar to the non-challenged control group by neutralizing the negative effects of the coccidial challenge.

## Introduction

Avian coccidiosis is a major parasitic disease that leads to significant intestinal tissue damage, higher mortality, and ineffective nutrient absorption resulting in economic losses to the poultry industry (Abdelrahman et al. 2014; Ott et al. 2018). Different *Emeria* species (belonging to phylum Apicomplexa) are responsible for avian coccidiosis (Quiroz-Castañeda and Dantán-González 2015). These are universal pathogens present in the poultry farm environment causing hindrance in the control of the disease (Abdelrahman et al. 2014). These parasites enter the mucosal membrane of the gastrointestinal tract (GIT), causing intestinal lesions, dehydration, and bloody diarrhea (Pattison et al. 2007; Yang et al. 2019). In broilers, these coccidial lesions act as a predisposing factor for necrotic enteritis (NE). The worldwide economic losses linked with NE in broilers, such as increased condemnations and reduced growth performance, are estimated to be six billion USD (Wade and Keyburn 2015). There are various antibiotic growth promoters (AGPs) and anticoccidial drugs commonly used to control coccidiosis, as well as secondary bacterial infection, that have been under scrutiny (Ritzi et al. 2014). Besides the prevention and treatment capability of these chemotherapeutic agents for controlling intestinal diseases, public concerns are on the rise regarding drug residues in poultry meat. Due to the higher prevalence of drug resistance these drugs are restricted as feed additives in poultry (Abdelrahman et al. 2014; Calik and Ergün 2015). Thus, the poultry industry and farmers focus on suitable alternatives to antibiotics and anticoccidial drugs to enhance bird performance, intestinal health, and promote healthy microbiota.

Vaccination is considered the best and common approach to prevent coccidiosis in modern poultry production systems. Live coccidia vaccines induce adaptive immunity. It leads to localized inflammation by causing damage to the intestinal epithelium (Williams 2002; Dalloul and Lillehoj 2005; Stringfellow et al. 2011). This trauma has been associated with reduced growth performance (Li et al. 2005). As a suitable alternative to antibiotics, organic acids (OAs) have favorable effects on intestinal health and birds’ performance and are effective for feed and food preservation (Rathnayake et al. 2021). Additionally, the OAs improve the growth performance and health of young broilers (Dibner and Buttin 2002; Pham et al. 2020). OAs have been reported as the best supplement for swine and poultry production by enhancing physiological functions, boosting the immune system, protecting GIT, modifying microbiota, and reducing the pH of GIT (Suiryanrayna and Ramana 2015; Khan and Iqbal 2016; Dittoe et al. 2018; Hamid et al. 2018). The OAs, including short-chain fatty acids (SCFAs), can substitute AGPs from broiler diets (Scicutella et al. 2021). The SCFAs, majorly propionic acid, acetic acid, and butyric acid, are produced by the fermentation of the carbohydrates performed by beneficial intestinal bacteria (Rawi et al. 2021).

Most importantly, OAs inclusion in feed and water can inhibit pathogenic bacteria, competing for nutrients with the host, and reduce the toxic metabolites of bacteria. OAs supplementation has the potential to retard the growth of pathogenic and zoonotic bacteria, e.g., *Salmonella* and *E. coli*, in the feed and GIT of birds. This leads to a positive impact on the health of birds and improved performance (Nguyen et al. 2020). OAs in their undissociated forms can pass through the cell membrane of bacteria. After entering the cell, OAs dissociate due to an alkaline environment of cytoplasm and produce H^+^ ions; bacterial cytoplasm becomes acidic. Bacteria try to restore the basic nature of cytoplasm by using its energy. However, the dissociate OA also produces anions (RCOO^−^) that can disrupt protein synthesis. Consequently, bacteria are unable to replicate due to cytoplasmic acidification. The cytoplasmic acidification, with the following uncoupling the creation and regulation of energy, was found to be a principal mechanism of OA for inhibiting pathogenic bacteria. The accumulation of anions in the cytoplasm of bacteria up to toxic levels has already been investigated *in-vitro* (Mani-López et al. 2012). The beneficial effects of OAs can be improved using blends rather than a single acid in broiler chickens (Polycarpo et al. 2017). Recently, proprietary commercial blends, as a water and feed additive of OAs, have been tested and developed in broiler chickens reared without antibiotics.

Currently, our laboratory works on OAs supplementation in broilers and found improved performance, intestinal integrity, microbial community, and antioxidative capacity in non-challenged trials. The effect of OAs supplementation, on broilers challenged with coccidia, has been seldom investigated. Therefore, the hypothesis for this study states that the coccidia challenge would cause intestinal damage, reduce immunity and performance. However, blend of selected OAs i.e., Formic acid, Acetic acid, and Ammonium Formate, (Trouw Nutrition, The Netherlands) as water additive, or blend of encapsulated butyrates, encapsulated MCFAs, organic acids (mainly sorbic acid), and phenolic compound (Trouw Nutrition, The Netherlands) as feed additive would partially recover or reduce intestinal lesions and improve intestinal integrity in broilers. The present experiment aimed to examine the effect of OAs supplementation in water or feed on performance, intestinal morphology, intestinal lesion scores, microbiota, and immunity parameters of broiler chickens exposed to experimental coccidia challenge.

## Materials and methods

### Birds, diets, and management

The present study was accomplished following guidelines of the standard recommendations of the National Institutes of Health for the Care and Use of Laboratory Animals. The current study protocol was approved (Ethical Code: SICAUAC201710-7) by the Animal Care and Use Committee of Sichuan Agricultural University, China.

A total of 360-day-old broiler chicks (Ross 308) obtained from a local commercial hatchery (Yuguan Co. Ltd., Chengdu city, Sichuan province, China), were allocated into six groups in 2 (Coccidial challenge or not) × 3 (dietary treatments) factorial design. The three dietary treatments included the control diet, the control diet plus the OAs in the drinking water, and the control diet plus OAs in the feed. The broilers were reared in the house, which was environmentally controlled, on cage pens at broiler farms, Sichuan Agricultural University, Yaan, China. This experiment was a completely randomized block study containing three dietary treatments with and without coccidial challenge and comprises a total of 6 treatments with 6 replicates of 10 birds per replicate. Broilers were randomly assigned to the treatments with two phase-feeding programs (Starter Phase: Day 1—21, Grower Phase: Day 22–35). The basal diet was formulated as a corn-soybean-based diet according to the NRC (1994) nutrient recommendations (Table 1). Experimental treatments comprised as follow; CON: Control diet without Coccidial Challenge; OAW: Organic acid in water without Coccidial Challenge; OAF: Organic acid in feed without Coccidial Challenge; CONC: Control diet with Coccidial Challenge; OAWC: Organic acid in water with Coccidial challenge; and OAFC: Organic acid in feed with Coccidial Challenge. All the diets were processed in mash form. Feed and water were provided to broilers ad libitum. Bird management was done as described in the Ross 308 Broiler Commercial Management Guide (Aviagen 2018).

### Coccidia challenge

On the 8th day **D**, the CON, OAW, and OAF groups were given (orally gavaged) 1 mL sterile water per bird, and the CONC, OAWC, and OAFC groups were orally gavaged with 50 times of the commercial attenuated vaccine (1 mL per bird) containing live attenuated oocysts of *Eimeria ****E**** acervulina*, *E. maxima*, and *E. tenella* (Foshan Standard Bio-Tech Co., Ltd., Foshan, China) by adopting the method as previously described by Wu et al. (2018).

### Materials preparation

Water acidifier (a blend of formic acid, acetic acid, and ammonium formate) was supplemented through drinking water (1.5 ml per 1L) during the whole experimental period. The inclusion of water acidifiers reduces the water pH from 7.56 ± 0.04 to 3.49 ± 0.05. The OAs blend as a feed additive (blend of encapsulated butyrate, encapsulated MCFAs, organic acids mainly sorbic acid, and phenolic compound) was added to the basal diets at 0.15% and 0.1% in Starter Phase (1–21 days) and Grower Phase (22–35 days), respectively.

**Table 1:** Formulation of basal diet fed by broilers during both stater and growth phase

Ingredients (%)	Starter phase	Grower phase
Corn	56.99	59.12
Soybean Meal (CP = 43%)	36.7	34
Soybean Oil	2.6	3.4
Limestone (CaCO_3_)	1.1	0.93
Calcium hydrogen phosphate	1.4	1.33
NaCl	0.4	0.4
Choline chloride	0.15	0.15
Multi-vitamins^1^	0.03	0.03
Mineral premix^2^	0.2	0.2
Lysine HCl (99%)	0.18	0.2
DL-methionine (99%)	0.25	0.24
Total	100	100
Nutrient level (Calculated)		
ME (kcal/kg)	2950	3020
CP (%)	21.03	20.07
Calcium (%)	1	0.9
Available phosphorus (%)	0.45	0.43
Lysine (%)	1.15	1.1
Methionine (%)	0.5	0.48
Methionine + Cysteine (%)	0.85	0.81

^1^Vitamin premix per kilogram feed provided: Vitamin A, 16,000 IU (trans retinol); Vitamin D3, 4,000 IU; Vitamin E, 1 IU (dl-α-tocopheryl acetate); Vitamin B1, 0.8 mg; Vitamin B2, 6.4 mg; Vitamin B12, 0.012 mg; Vitamin B6, 2.4 mg; calcium pantothenate, 10 mg; niacin acid, 14 mg; biotin, 0.1 mg; folic acid, 0.2 mg; Vitamin K3, 2 mg

^2^Mineral premix per kilogram feed provided: Fe (FeSO4·H2O), 100 mg; Cu (CuSO4·5H2O), 12.5 mg; Mn (MnSO4·H2O), 88 mg; Zn (ZnSO4·H2O), 95 mg; I (KI), 0.9 mg; Se (Na2SeO3), 0.3 mg

### Growth performance

All broilers in each pen, after 12 h fasting, body weight (BW), and the amount of feed intake (FI; offered – remained) by pen were measured at the end of each phase (starter and grower) on 21 d and 35 d. Average body weight (ABW), average body weight gain (ABWG), average feed intake (AFI) and feed conversion ratio (FCR) by pen were calculated. Birds were monitored to account for their morbidity (health status) and mortality, on daily basis. During the experiment, dead birds were weighed, and mortality was included during growth performance calculations.

### Sample collections

On d 18 and 29, after 12 h fasting, two broilers from each replicate (12 from each treatment), with BW near to the ABW of the pen, were selected and blood samples were obtained from a jugular vein before slaughter. The blood samples were centrifuged (2000×*g*, 10 min, 4 °C) to obtain plasma, and stored at − 20 °C for future analysis. After plasma samples were collected from twelve birds, all selected birds were slaughtered by severing their jugular vein and dissected to the gross examination of jejunum for lesion score. After gross examination, jejunum (from six birds) was stored in a 4% paraformaldehyde solution of histological analysis. However, the other six birds were used for cecal tonsil and jejunum mucosa flash-frozen in liquid nitrogen − 80 ℃ until gene expression analysis. Afterward, ileal chyme samples were collected and stored at − 80 ℃ for microbial community analysis.

### Plasma analysis

Plasma tumor necrosis factor-α (TNF-α), tumor necrosis factor-γ (TNF-γ), interleukin 2 (IL-2), interleukin 10 (IL-10), immunoglobulin M (IgM), immunoglobulin G (IgG), and immunoglobulin A (IgA) were determined using the enzyme-linked immunosorbent assay (ELISA) kits that were purchased from Jiangsu Jingmei Biological TechnologyCo., Ltd. (Yancheng, Jiangsu, China) and followed the procedure of the instruction of kit.

### Intestinal characteristics

#### Jejunum lesion score

On 18 d and 29 d, 12 chickens from each treatment group were slaughtered and jejunal lesions were scored (Johnson and Reid 1970) by using the following scale: Score 0: there will be no gross lesions on jejunum. Score 1: at the middle of the jejunum, small red petechiae may present on the serosal side. There is no thickness or ballooning of the jejunum, however, small quantities of orange color mucus might be present inside the jejunum. Score 2: higher number of petechiae present on the serosal surface, a large quantity of orange mucus present in the jejunum, little or absence of the thickness or ballooning of jejunum. Score 3: The presence of a thick and ballooned intestinal wall. Rough mucosal surface and contents of jejunum contain mucus and pinpoint clots of blood. Score 4: the most of jejunal part becomes ballooned, includes a higher amount of red blood cells and blood clots, and the jejunal wall becomes thicker.

#### Jejunum morphology and counting of goblet cell

Six samples from each treatment of fixed jejunal segments in 4% neutral buffered paraformaldehyde solution were rinsed in ethyl alcohol and embedded in paraffin wax. The samples were cut (5 µm) using a propeller slicer (Leica-2016, Germany), with 3 slices per treatment, and stained using the hematoxylin and eosin method. A microscope (BA400Digital, Motic China Group Co., LTD, China) was used to take the micrograph. Measurements were performed for villus height (VH), crypt depth (CD), and calculations were made for the ratio of villus height to crypt depth (VH:CD), the count of the goblet cells (GC), and the number of GCs per unit area by using Image-Pro Plus 6.0 (Media Cybernetics, USA) for each structure per slice. The tip of the villus to the villus-crypt junction was defined as VH. In contrast, the CD was measured from the depth of the invagination to adjacent villi (Han et al. 2017).

#### Jejunum mucosal and cecal tonsil mRNA gene expression

On 18 d and 29 d, six samples from each treatment of jejunal mucosa and cecal tonsil segment were subjected to mRNA gene expression analysis. Total RNA was extracted by using a TRIzol reagent kit (Takara, Dalian, China), and synthesis of cDNA was completed by using the reagent kit (PrimeScript RT, Takara). Then, Real-time quantitative PCR was performed in triplicate on a QuantStudio 6 Flex system (Applied Biosystems, Foster City, CA) using an SYBR Premix Ex Taq II kit (No. RR820A, Takara, Dalian, China) as per the manufacturer’s instructions (Wu et al. 2019). Primers for Tight Junction Protein (TJP), 3 genes in association with the intestinal barrier including Claudin1 (CLDN1), Zonula Occludens-1 (ZO-1), Occludin (OCLN), and β-actin (housekeeping gene) were designed using Primer Express 3.0 (Applied Biosystems; Table 2). The expression level of RNA is quantified using the ^2−ΔΔ^Ct equation according to Livak and Schmittgen, (2001).

**Table 2:** Primers used for the quantitative RT-PCR of the target genes

Target gene	Forward/reverse sequence (5′ to 3′)	Gen Bank Accession no	References
β-actin	F: TTGGTTTGTCAAGCAAGCGG	NM_205518.1	Li et al. (2019)
	R: CCCCCACATACTGGCACTTT		
Zonula Occludens-1	F: TGTAGCCACAGCAAGAGGTG	XM_413773	
	R: CTGGAATGGCTCCTTGTGGT		
Claudin-1	F: TGGAGGATGACCAGGTGAAGA	NM_001013611.2	Shao et al. (2013)
	R: CGAGCCACTCTGTTGCCATA		
Occludin	F: TCATCGCCTCCATCGTCTAC	NM_205128.1	
	R: TCTTACTGCGCGTCTTCTGG		

#### 16S rDNA gene amplicons analysis of ileal chyme

According to manufacturer instructions, at d 18 and 29, six ileal chyme samples from each treatment were subjected for extraction of DNA by using QIAamp PowerFecal DNA Kit (Qiagen, Hilden, Germany). The DNA concentration and quality were checked using a NanoDrop Spectrophotometer. Analysis of 16S rDNA gene amplicons was performed using the Novo gene platform (Illumina Hiseq, Novogene Bioinformation Technology, Beijing, China). All methods including extraction of DNA, 16 s rRNA sequencing, processing of sequences, and analysis of data were performed according to Qin et al. (2019). Concisely, sterile water was used to dilute DNA up to 10 ng/μL. The 16S rRNA genes of distinct regions (16S V4) were amplified using a specific primer (515F GTGCCAGCMGCCGCGGTAA; 806R GGACTACHVGGGTWTCTAAT) with the unique barcodes. The Phusion High-Fidelity PCR Master Mix (New England Biolabs) was used to carry out all PCR reactions. The mixture of PCR products was prepared by using equal density ratios. Then, Qiagen Gel Extraction Kit (Qiagen, Germany) was used to purify the mixture of products from PCR. According to manufacturer recommendations, sequencing libraries were generated using TruSeq DNA PCR-Free Sample Preparation Kit (Illumina, USA), and the addition of index codes was completed. The library quality was assessed on the Agilent Bioanalyser 2100 system and Qubit 2.0 Fluorometer (Thermo Scientific). Lastly, the eligible libraries were sequenced on an Illumina HiSeq 2500 platform and 250 bp paired-end reads were generated. Reads were filtered by QIIME quality filters. Sequences with ≥ 97% similarity were assigned to the same optimal taxonomic units (OTUs). The different taxonomical levels were examined by using the relative abundance of OTU. Diversity within communities, i.e., alpha diversity (Observed Species, Simpson, Shannon, Chao1, ACE, Good coverage, and Phylogenetic Distance) calculations and taxonomic community assessments were performed by QIIME 1.7.0, and Beta diversity included weighted Unifrac distances calculated with 10 times subsampling, and distances were visualized by principal coordinate analysis (PCoA), and the separation was tested using R in ANOSIM (Lozupone and Knight 2005). The sequence data have been deposited in the NCBI Sequence Read Archive database (Accession No. PRJNA745096).

### Statistical analysis

The experiment was a completely randomized design with cages were considered as experimental units. Performance, plasma immune indices, intestinal morphology, mRNA data, and ileal microbiota analysis were analyzed by two-way ANOVA to determine the main effects (supplementation of OAs in water or feed and coccidia challenge) and their interaction using the generalized linear model (GLM) procedure of SAS 2004 model. For plasma immune indices, intestinal morphology and intestinal lesion score the randomly selected birds were the experimental unit. For intestinal lesion score results were analyzed by a one-way analysis of variance (ANOVA) using the GLM procedure of SAS (SAS Institute; Cary, NC). Differences among means were tested with Duncan’s multiple range tests. A probability value of *P* < 0.05 was statistically significant.

## Results

### Growth performance

The effect of supplementation of OAs in water or feed with and without coccidial challenge on growth performance, i.e., ABW, ABWG, AFI, and FCR, are summarized in Table 3. The results indicated that supplementation of OAs in water or feed improved (P < 0.01) the ABW, ABWG, and FCR compared with the control diet during starter, grower, and whole experimental period. Coccidial challenge decreased ABW, ABWG, and AFI, and increased FCR during the starter and whole experimental period (P < 0.05). Although there was no interaction between OAs supplementation and coccidial challenge, the supplementation had improved the overall performance with and without coccidial challenge birds on 21 d and 35 d. The FI was not significantly affected by the OAs supplementation and its interaction with the coccidial challenge.

**Table 3:** Effect of organic acids on the growth performance of broilers with and without oral coccidial challenge

Treatments^1^	C.C^2^	ABW^3^			ABWG^3^			AFI^3^			FCR^3^			Mortality		
		1d	21d	35d	1–21d	22–35d	1–35d	1–21d	22-35d	1-35d	1-21d	22-35d	1-35d	1-21d	22-35d	1-35d
		g			g/chick			g/chick			g/g			%		
CON	−	34.8	742	1642	707	900	1607	978	1542	2478	1.38	1.73	1.55	0.0	2.08	1.67
OAW	−	34.8	785	1855	750	1070	1820	990	1715	2665	1.32	1.61	1.47	0.0	0.00	0.00
OAF	−	34.8	804	1831	769	1027	1797	1012	1695	2658	1.32	1.65	1.48	0.0	0.00	0.00
CONC	+	34.9	667	1561	632	894	1526	902	1634	2498	1.44	1.83	1.64	0.0	0.00	0.00
OAWC	+	34.8	723	1682	688	959	1647	917	1577	2445	1.34	1.64	1.49	1.67	0.00	1.67
OAFC	+	34.9	719	1715	684	997	1680	929	1634	2520	1.36	1.64	1.50	1.67	0.00	1.67
SEM		0.06	14.46	41.73	14.46	33.5	41.73	14.60	55.35	61.95	0.016	0.038	0.022	0.962	0.851	0.179
Main effect means																
CON		34.8	704^b^	1601^b^	670^b^	897^b^	1566^b^	940	1588	2488	1.41^a^	1.78^a^	1.59^a^	0.00	1.04	0.83
OAW		34.8	754^a^	1768^a^	719^a^	1014^a^	1733^a^	953	1646	2555	1.33^b^	1.63^b^	1.48^b^	0.83	0.00	0.83
OAF		34.8	761^a^	1773^a^	726^a^	1012^a^	1738^a^	970	1665	2589	1.34^b^	1.64^b^	1.49^b^	0.83	0.00	0.83
C.C	−	34.8	777^a^	1776^a^	742^a^	999	1741^a^	993^a^	1651	2600^a^	1.34^b^	1.66	1.49^b^	0.00	0.69	0.56
	+	34.8	703^b^	1652^b^	668^b^	950	1618^b^	916^b^	1615	2488^b^	1.38^a^	1.70	1.54^a^	1.11	0.00	1.11
*P-Value*	Diet	0.806	0.001	0.0003	0.001	0.002	0.0003	0.130	0.361	0.270	< 0.0001	0.001	< 0.0001	0.612	0.380	1.000
	C.C	0.393	< 0.0001	0.001	< 0.0001	0.082	0.001	< 0.0001	0.436	0.034	0.008	0.194	0.017	0.168	0.325	0.568
	Diet × C.C	0.849	0.730	0.550	0.729	0.283	0.550	0.935	0.123	0.160	0.534	0.342	0.182	0.612	0.380	0.279

^1^*CON* Control diet, *OAW* Organic acid in water, *OAF* Organic acid in feed, *CONC* Control diet with Coccidial challenge, *OAWC* Organic acid in water with Coccidial challenge, and *OAFC* Organic acid in feed with Coccidial challenge

^2^C.C = Coccidial Challenge

^3^Average body weight (ABW); Average body weight gain (ABWG); Average feed intake (AFI); Feed conversion ratio (FCR)

^a, b^Mean within each column with no common superscript differ significantly (P < 0.05)

### Plasma immune indices

The effect of supplementation of OAs in water or feed with and without coccidial challenge on plasma immune indices is represented in Table 4. Results showed no interaction of OAs supplementation and coccidial challenge for plasma immune indices on 18 d and 29 d, except IgG was found higher (P = 0.03) in OAF groups as compared with all other groups. OAs supplementation in feed increased TNF-γ on 18 d compared to other groups (P = 0.006). In contrast, all other immune indices were found similar by OAs supplementation in water or feed and control diet. On 18 d, the coccidial challenge decreases TNF-γ (P = 0.01) and IL-10 (P =  < 0.0001), and increases IgM (P = 0.03), IgG (P = 0.04) and IgA (P = 0.02), however TNF-α and IL-2 found similar among treatments. On 29 d, the coccidial challenge increases IgM and IgA, whereas TNF-α, TNF-γ, IL-2, IL-10, and IgG were found similar among all groups.

**Table 4:** Effect of organic acids on serum immune indices of broilers with and without coccidial challenge

Treatments^1^	C.C^2^	Mearsurements^3^													
		TNF-α		IFN-γ		IL-2		IL-10		IgM		IgG		IgA	
		18d	29d	18d	29d	18d	29d	18d	29d	18d	29d	18d	29d	18d	29d
		pg /ml								µg/ml					
CON	−	65.8	55.3	68.0	59.0	297.1	231.1	55.7^b^	55.8	841.4	837.8	2687.8^b^	2565.6	1735.9	1795.8
OAW	−	61.1	50.0	77.2	61.2	289.6	214.8	59.4^b^	48.7	790.4	782.6	2534.9^b^	2148.3	1725.5	1611.8
OAF	−	58.7	65.4	71.7	67.0	259.0	243.0	77.4^a^	50.6	740.5	873.7	2010.6^b^	2254.3	1575.7	1832.3
CONC	+	83.1	63.0	61.5	62.2	352.7	272.6	58.6^b^	52.0	1070.7	1081.6	2708.6^b^	2621.2	2222.4	2393.7
OAWC	+	71.7	53.5	68.9	57.4	272.7	270.8	50.0^b^	49.4	812.9	971.3	2659.7^b^	2357.2	2093.0	2186.9
OAFC	+	77.6	54.2	54.3	76.7	417.2	252.6	37.5^c^	56.9	1595.6	955.5	3985.1^a^	2141.4	2468.8	1963.1
SEM		10.4	4.77	4.83	3.94	53.20	21.90	3.89	4.13	202.8	68.2	409.7	220.8	298.1	194.7
Main effect means															
CON		74.4	59.2	64.8	60.6^b^	324.9	251.9	57.1	53.9	956.0	959.7	2698.2	2593.4	1979.1	2094.7
OAW		66.4	51.7	73.1	59.3^b^	281.2	242.8	54.7	49.0	801.6	876.9	2597.3	2252.7	1909.2	1899.3
OAF		68.1	59.8	63.0	71.9^a^	338.1	247.8	57.4	53.8	1168.0	914.6	2997.8	2197.9	2022.2	1897.7
C.C	−	61.9	56.9	72.3^a^	62.4	281.9	229.6	64.2^a^	51.7	790.7^b^	831.3^b^	2411.1^b^	2322.7	1679.0^b^	1746.6^b^
	+	77.4	56.9	61.6^b^	65.4	347.5	265.3	48.7^b^	52.8	1159.7^a^	1002.8^a^	3117.8^a^	2373.3	2261.4^a^	2181.2^a^
*P-value*	Diet	0.722	0.188	0.101	0.006	0.54	0.917	0.794	0.418	0.21	0.487	0.602	0.17	0.93	0.516
	C.C	0.076	0.992	0.011	0.352	0.141	0.055	< 0.0001	0.755	0.034	0.004	0.043	0.781	0.023	0.01
	Diet × C.C	0.915	0.13	0.483	0.25	0.269	0.562	< 0.0001	0.48	0.119	0.49	0.039	0.769	0.769	0.412

^1^*CON* Control diet, *OAW* Organic acid in water, *OAF* Organic acid in feed, *CONC* Control diet with Coccidial challenge, *OAWC* Organic acid in water with Coccidial challenge, *OAFC* Organic acid in feed with Coccidial challenge

^2^C.C = Coccidial Challenge

^3^Tumor necrosis factor-alpha (TNF-α); Tumor necrosis factor-gamma (TNF-γ); Interleukin-2 (IL-2); Interleukin-10 (IL-10); Immunoglobulin M (IgM); Immunoglobulin G (IgG); and Immunoglobulin A (IgA)

^a−c^Mean within each column with no common superscript differ significantly (P < 0.05)

### Intestinal characteristics

#### Jejunum lesion score

**Fig. 1:**
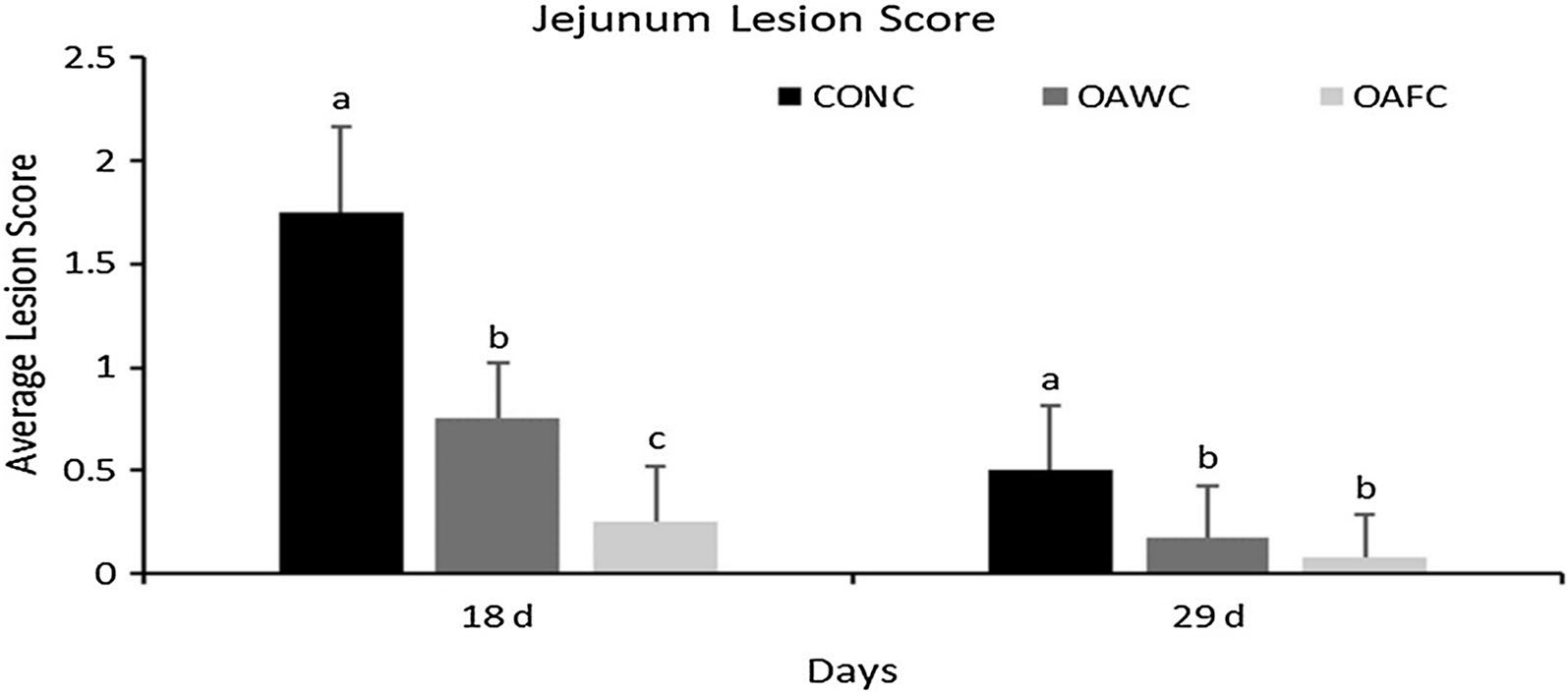
Effect of Organic Acids in water or feed with the coccidial challenge on Jejunal lesion score on 18 d and 29 d. Each bar represents the Mean ± SE value (N = 12)*.* *CONC* Control diet with Coccidial challenge, *OAWC* Organic acid in water with Coccidial challenge, and *OAFC* Organic acid in feed with Coccidial challenge. ^a−c^ Mean within each bar graph with no common superscript differ significantly (P < 0.05)

Lesions in the jejunum observed on 18 d and 29 d are represented in Fig. 1. There was no lesion found in birds belong to non-challenged groups. Whereas on 18 d, jejunal lesion score was found significantly higher in CONC groups than OAWC and OAFC groups. Moreover, lesion score was significantly lower in OAFC groups from both groups (P < 0.0001). On 29 d, a similar trend was observed, i.e., lesion score was found significantly higher in CONC groups than OAWC and OAFC groups (P = 0.03). These results indicated that supplementation of OAs, in water or feed in coccidial challenged groups, can improve intestinal health, and reduces intestinal lesions.

#### Jejunum morphology and counting of goblet cell

**Fig. 2:**
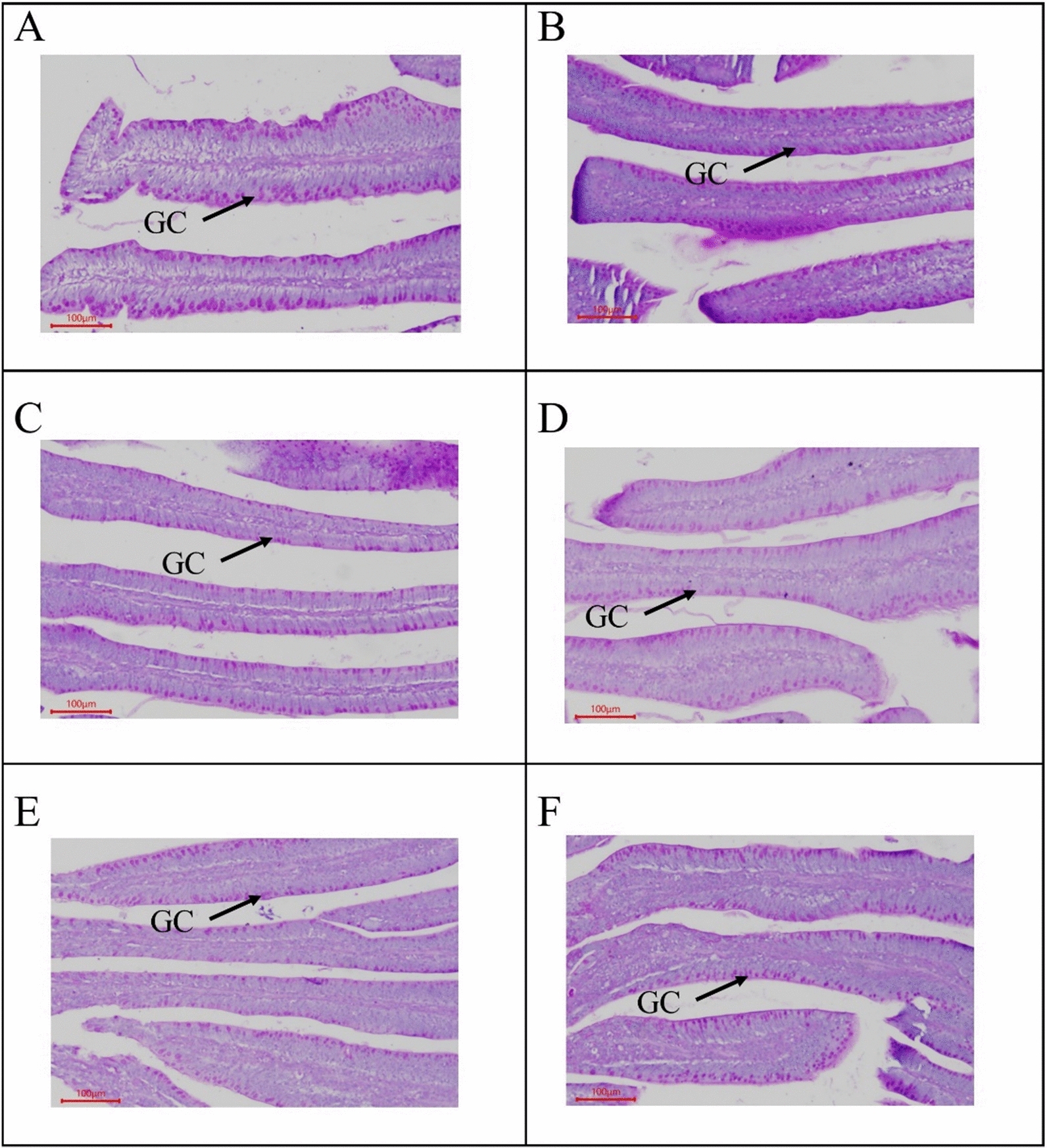
Jejunum villus morphology on 18 d (N = 6). GC: goblet cells. **A** *CON* Control diet; **B** *OAW* Organic acid in water; **C** *OAF* Organic acid in feed; **D** *CONC* Control diet with Coccidial challenge; **E** *OAWC* Organic acid in water with Coccidial challenge; and **F** *OAFC* Organic acid in feed with Coccidial challenge

**Fig. 3:**
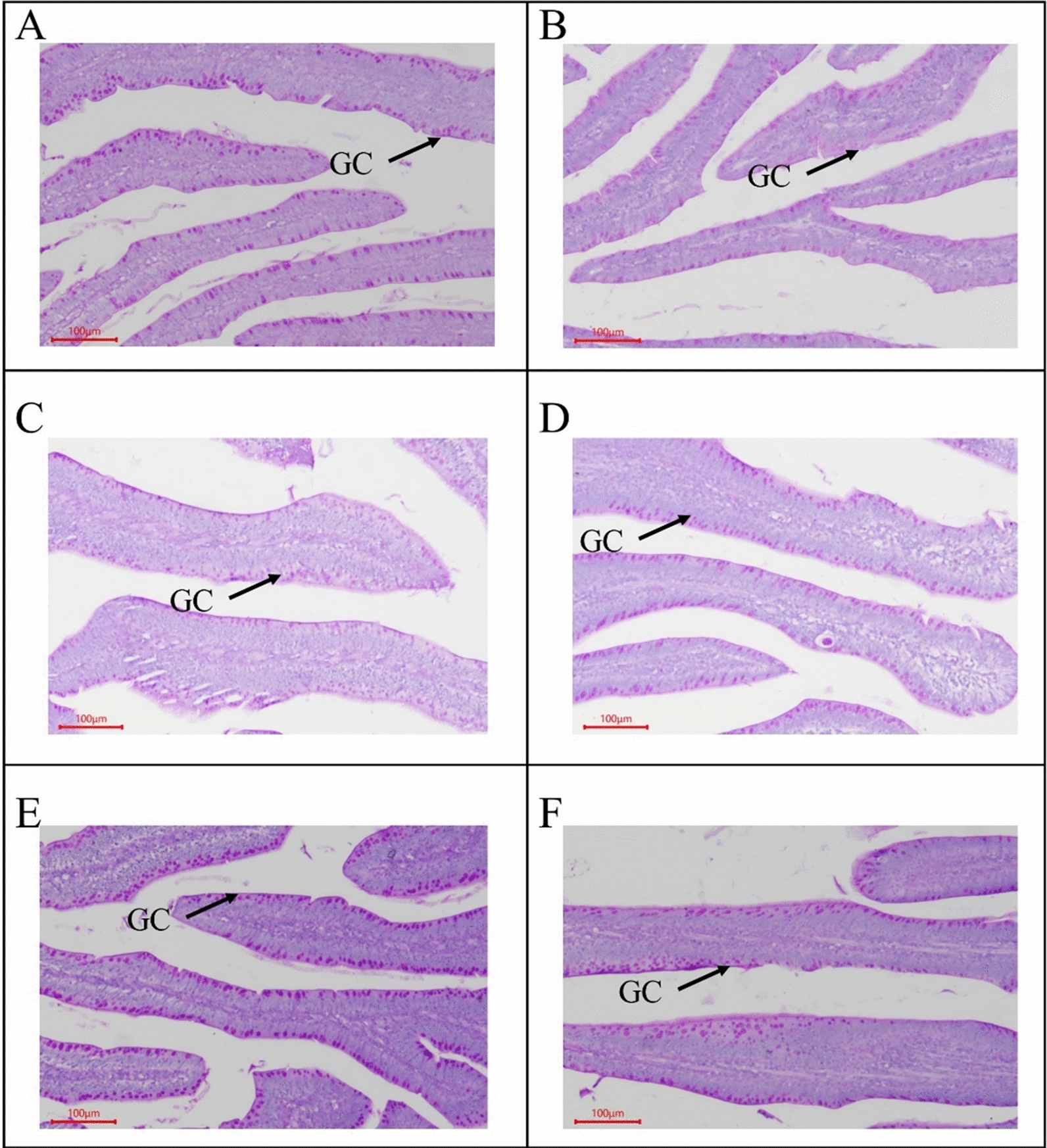
Jejunum villus morphology on 29 d (N = 6). GC: goblet cells. **A** *CON* Control diet; **B** *OAW* Organic acid in water; **C** *OAF* Organic acid in feed; **D** *CONC* Control diet with Coccidial challenge; **E** *OAWC* Organic acid in water with Coccidial challenge; and **F** *OAFC* Organic acid in feed with Coccidial challenge

On 18 d and 29d, jejunal morphological i.e., VH, CD, VH:CD, and GC, results indicated that there was no interaction observed between OAs supplementation and coccidial challenge, as well as no effect was observed by the OAs supplementation on jejunal morphology (Table 5). VH was not affected by coccidial challenge, whereas CD was found higher, and VH:CD was found lower in the coccidial challenge group as compared to the non-challenge group on 18 and 29 d. However, the GC was found higher in the non-coccidial challenge on 18 d (Fig. 2), however no difference between the challenged and non-challenged group on 29 d (Fig. 3).

**Table 5:** Effect of organic acids on intestinal morphology of broilers with and without coccidial challenge

Treatments^1^	C.C^2^	Measurements^3^							
		VH		CD		VH:CD		GC	
		18d	29d	18d	29d	18d	29d	18d	29d
		µm				µm/µm		NO./mm^2^	
CON	−	1077.5	1269.4	176.3	213.8	6.35	6.00	1983.6	1727.8
OAW	−	1087.6	1181.3	190.2	220.0	5.95	5.41	1786.0	1470.8
OAF	−	1082.4	1191.0	167.6	197.4	7.13	6.23	1866.5	1609.4
CONC	+	1273.7	1014.0	247.8	257.7	5.29	4.06	1514.1	1831.8
OAWC	+	1084.0	1168.5	237.8	251.9	4.72	4.77	1718.7	1716.4
OAFC	+	998.4	1200.9	217.1	255.5	4.86	4.78	1511.5	1710.7
SEM		75.5	81.6	27.6	20.6	0.59	0.40	106.0	159.2
Main effect means									
CON		1175.6	1141.7	212.0	235.8	5.82	5.03	1748.8	1779.8
OAW		1085.8	1174.9	214.0	236.0	5.34	5.09	1752.4	1593.6
OAF		1040.4	1195.9	192.3	226.4	6.00	5.50	1689.0	1660.1
C.C	−	1082.5	1213.9	178.0^b^	210.4^b^	6.48^a^	5.88^a^	1878.7^a^	1602.7
	+	1118.7	1127.8	234.2^a^	255.0^a^	4.96^b^	4.54^b^	1581.4^b^	1753.0
*P-value*	Diet	0.211	0.801	0.691	0.871	0.519	0.453	0.799	0.505
	C.C	0.562	0.209	0.02	0.014	0.004	0.001	0.002	0.259
	Diet × C.C	0.183	0.218	0.892	0.818	0.547	0.284	0.17	0.875

^1^*CON* Control diet, *OAW* Organic acid in water, *OAF* Organic acid in feed, *CONC* Control diet with Coccidial challenge, *OAWC* Organic acid in water with Coccidial challenge; and *OAFC* Organic acid in feed with Coccidial challenge

^2^C.C = Coccidial Challenge

^3^Villus height (VH); Crypts Depth (CD); Villus Height to Crypt depth ratio (VH:CD); and Goblet cell (GC)

^a, b^Mean within each column with no common superscript differ significantly (P < 0.05)

#### Jejunum mucosal and cecal tonsil mRNA gene expression

On 18 d and 29d, jejunal mucosal and cecal tonsil mRNA i.e., CLDN1, ZO-1, and OCLN results indicated that there was no interaction observed between OAs supplementation and coccidial challenge, as well as no effect was observed by the OAs supplementation, and coccidial challenge alone on jejunum mucosal and cecal tonsil mRNA genes expressions (Table 6).

**Table 6:** Effect of organic acids on mRNA gene expression in the jejunum and cecal tonsil of broilers with and without coccidial challenge

Treatments^1^	C.C^2^	Jejunum^3^						Cecal Tonsil^3^					
		18d			29d			18d			**29d**		
		*CLDN1*	*ZO-1*	*OCLN*	*CLDN1*	*ZO-1*	*OCLN*	*CLDN1*	*ZO-1*	*OCLN*	*CLDN1*	*ZO-1*	*OCLN*
CON	−	1.06	1.14	1.29	1.32	1.14	1.06	1.02	1.09	1.13	1.04	1.01	1.05
OAW	−	1.49	2.43	3	1.89	1.4	1.81	2.3	1.45	1.34	1.31	1.41	1.23
OAF	−	1.11	1.06	0.72	1.94	1.93	4.03	1.94	2	1.96	0.61	1.04	1.27
CONC	+	1.22	1.24	1.13	0.92	1.22	2.39	1.1	1.35	2.39	0.38	0.71	1.51
OAWC	+	2.04	1.04	0.8	0.97	2.02	4.31	1.52	1.73	1.47	0.83	1.22	1.24
OAFC	+	2.13	1.26	1.43	3.41	0.98	2.66	1.06	1.49	1.52	0.69	0.92	0.87
SEM		0.65	0.591	0.836	0.775	0.365	1.538	0.557	0.304	0.337	0.26	0.225	0.221
Main effect means													
CON		1.14	1.19	1.21	1.12	1.18	1.73	1.06	1.22	1.76	0.71	0.86	1.28
OAW		1.77	1.73	1.9	1.43	1.71	3.06	1.91	1.59	1.41	1.07	1.32	1.23
OAF		1.62	1.16	1.08	2.67	1.45	3.34	1.5	1.75	1.74	0.65	0.98	1.07
C.C	−	1.22	1.54	1.67	1.72	1.49	2.3	1.75	1.52	1.48	0.99	1.15	1.18
	+	1.8	1.18	1.12	1.77	1.41	3.12	1.23	1.52	1.79	0.63	0.95	1.21
*P-value*	Diet	0.61	0.559	0.58	0.128	0.365	0.54	0.331	0.227	0.516	0.235	0.13	0.623
	C.C	0.288	0.459	0.432	0.939	0.791	0.519	0.259	0.977	0.262	0.109	0.28	0.901
	Diet × C.C	0.802	0.338	0.223	0.287	0.114	0.446	0.641	0.357	0.054	0.358	0.926	0.167

^1^*CON* Control diet, *OAW* Organic acid in water, *OAF* Organic acid in feed, *CONC* Control diet with Coccidial challenge, *OAWC* Organic acid in water with Coccidial challenge; and *OAFC* Organic acid in feed with Coccidial challenge

^2^C.C = Coccidial Challenge

^3^*CLDN1* = Claudin1; *ZO-1* = Zona Occludin-1; *OCLN* = Occludin

### Ileal microbial community

**Fig. 4:**
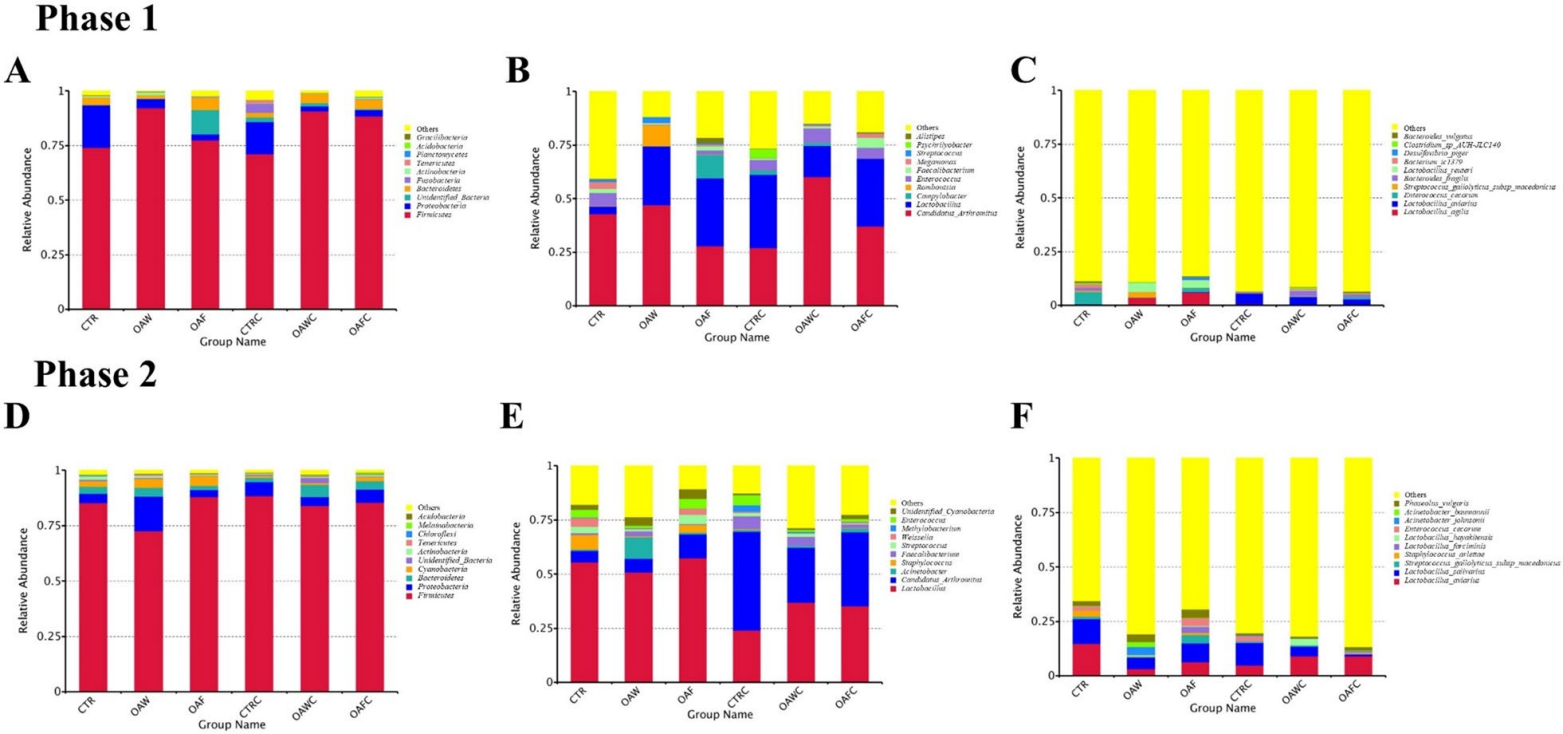
Taxonomic differences in the microbial community of the ileum in broilers on 18 and 29 d. Relative abundance levels of the bacterial (**A** & **D**) phyla, (**B** & **E**) genu and their (**C** & **F**) species are present in the six treatments. *CON* Control diet, *OAW* Organic acid in water, *OAF* Organic acid in feed, *CONC* Control diet with Coccidial challenge, *OAWC* Organic acid in water with Coccidial challenge, and *OAFC* Organic acid in feed with Coccidial challenge

The microbial communities were compared, in the ileum among six dietary groups, by using Illumina Hiseq High-throughput sequencing. On 18 d, a total of 2,122,845 sequencing reads were obtained from the ileal chyme samples. Through cutting and filtering of reads, an average of 73,202 reads was measured per sample, and an average of 69,049 valid data was obtained after quality control. The effective rate of quality control was 94.16%. The sequences were clustered into Operational Taxonomic Units (OUTs) with 97% identity, and a total of 1518 OTUs were obtained. In the annotation results, with the Silva132 database, there were 99.54, 98.54, 85.05, 78.46, 72.60, 48.48, and 16.86% OTU annotations proportion of kingdom, phylum, class, order, family, genera, and species level, respectively. The most abundant phyla were *Firmicutes*, *Proteobacteria*, and *unidentified_Bacteria*, and their abundance was not significantly different among all experimental groups including coccidial challenge and OAs alone, and their interaction (Fig. 4A). At the genus level including *Candidatus_Arthromitus*, *Lactobacillus*, and *Campylobacter* were found dominant but significantly similar in coccidial challenge groups, OAs supplemented groups, and their interaction (Fig. 4B). At the species level, dominant species were *Lactobacillus_agilis*, *Lactobacillus_aviarius*, and *Enterococcus_cecorum*. There was no significant interaction between OAs and coccidial challenge. Coccidia challenge decreases (P = 0.02) *Lactobacillus_reuteri* as compared to non-challenged group (Fig. 4C).

**Fig. 5:**
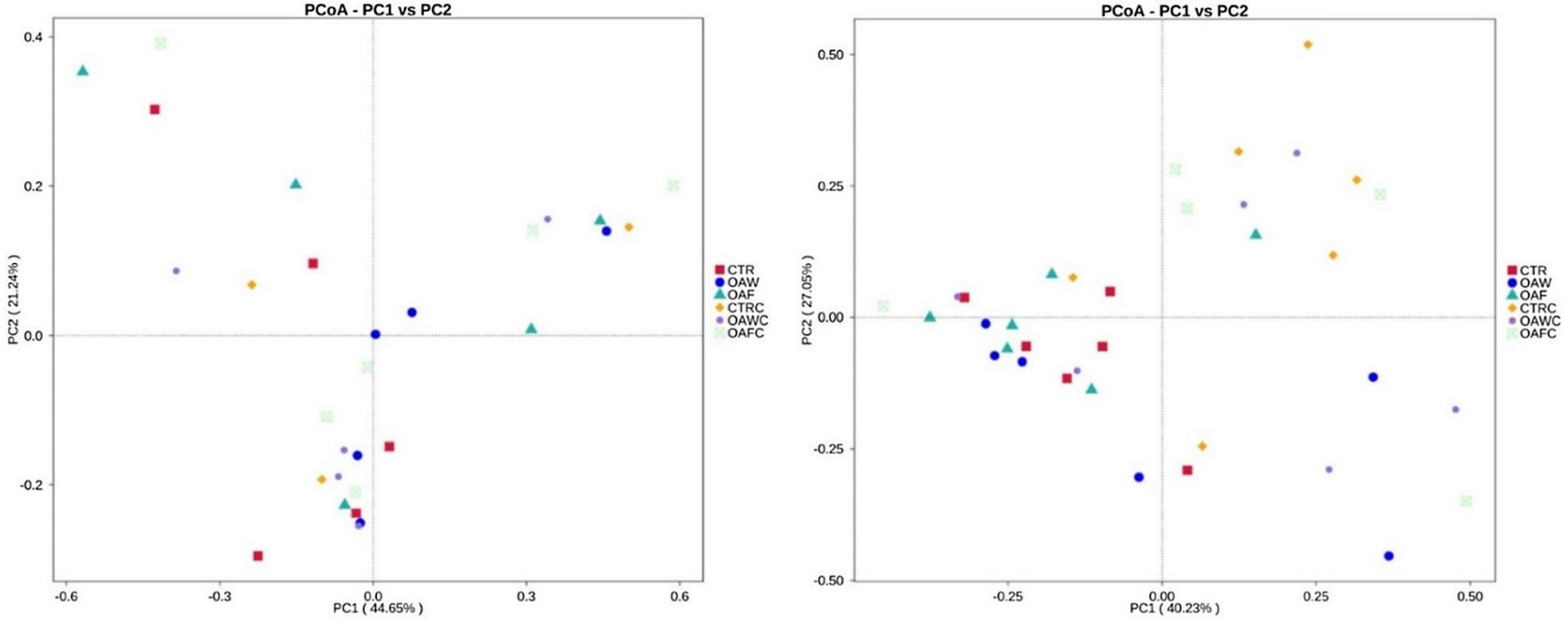
Beta (β) diversity analysis in the microbial community of the Ileal chyme in broilers. Weighted unifrac PCoA plot (based on OTUs) according to 6 treatments. (left) 18 d; (right) 29 d. PC1 and PC2 in x-and y-axis represented two principal discrepancy components between groups, and the percentage in bracket means contribution value to the discrepancies by the component. Color dots represent samples. Samples in the same group share the same color. *CON* Control diet, *OAW* Organic acid in water, *OAF* Organic acid in feed, *CONC* Control diet with Coccidial challenge, *OAWC* Organic acid in water with Coccidial challenge, and *OAFC* Organic acid in feed with Coccidial challenge

On 29 d, a total of 2,668,366 sequencing reads were obtained from the ileal chyme samples, and through cutting and filtering of reads, an average of 76,239 reads was measured per sample, and an average of 71,787 valid data was obtained after quality control. The effective rate of quality control was 94.18%. The sequences were clustered into OTUs with 97% identity, and a total of 1,879 OTUs were obtained. In the annotation results, with the Silva132 database, there were 100, 92.87, 86.85, 77.65, 70.84, 48.43, and 15.54% OTU annotations proportion of kingdom, phylum, class, order, family, genera, and species level, respectively. The most abundant phyla were *Firmicutes*, *Proteobacteria,* and *Bacteroidetes*. However, coccidial challenge alone, OAs supplementation, and their interaction had no significant effect on phylum level among all experimental groups (Fig. 4D), except *Cyanobacteria* abundance reduced (P = 0.014) in the challenged group as compared to the non-challenged group. At the genus level, dominant species were *Lactobacillus*, *Candidatus arthromitus*, and *Acinetobacter*. No significant interaction of OAs and coccidial challenge was found, and OAs supplementation also had a similar effect on genus species. However, *Lactobacillus* (P = 0.036) and *unidentified Cyanobacteria* (P = 0.01) were found higher in the non-challenged group than the coccidial challenge group. *Candidatus arthromitus* abundance was found higher in the coccidial challenged group than the non-challenged group (P = 0.0008; Fig. 4E). At the species level, dominant species were *Lactobacillus aviarius*, *Lactobacillus salivarius*, and *Streptococcus gallolyticus subsp macedonicus*. No significant interaction of OAs and coccidial challenge was found, and OAs supplementation also had a similar effect on the abundance of species. However, *Phaseolus vulgaris* was found higher in the non-challenged group than the coccidial challenged group (P = 0.01; Fig. 4F). Results of Alpha diversity, on d 18 and 29, are represented in Table 7. The diversity and richness within the microbial community were reflected by the Simpson, Shannon, ACE, and Chao1. ACE and Chao1 are used to indicate the richness within species, however the Simpson and Shannon indexes represent the diversity (evenness) of the microbial community in a population. During both d 18 and 29, the alpha diversity was not significantly affected as there was no interaction between OAs supplementation and coccidial challenge, as well as coccidial challenge and OAs alone had not significantly affected the alpha diversity, except Simpson was higher in the non-challenged group than coccidial challenged group (P = 0.02). Additionally, beta diversity metric was used to compare species diversity and abundance between samples, and this relationships between communities of various bacteria belong to different treatments were characterized by PCoA (weighted UniFrac), and the results exhibited that interaction of OAs and coccidial challenged groups did not influence microbial communities of ileal chyme after 18 d and 29 d (Fig. 5). However, after 29 d, coccidial challenge produces significant effect on the beta diversity (R = 0.296, P = 0.002).

**Table 7:** Effect of organic acids on alpha diversity of ileal chyme of broilers with and without coccidial challenge

Treatment^1^	C.C	Observed Species		Shannon		Simpson		Chao1		ACE		Good Coverage		Phylogenetic Distance	
		18d	29d	18d	29d	18d	29d	18d	29d	18d	29d	18d	29d	18d	29d
CON	−	317.2	387.7	3.69	5.12	0.73	0.91	401.3	424.1	403.8	427.9	0.995	0.998	45.60	44.70
OAW	−	274.8	497.0	3.51	5.25	0.78	0.90	339.9	568.4	362.3	571.8	0.995	0.996	29.01	53.28
OAF	−	296.6	363.3	3.82	4.67	0.76	0.90	325.4	431.2	337.9	424.5	0.997	0.997	52.46	41.80
CONC	+	280.3	379.8	3.83	3.86	0.84	0.75	351.5	453.8	357.4	470.6	0.996	0.996	36.11	42.05
OAWC	+	291.2	447.8	3.27	5.07	0.72	0.90	350.9	507.2	365.3	508.1	0.996	0.996	40.98	51.92
OAFC	+	355.2	374.0	4.06	4.30	0.76	0.83	431.3	465.7	423.0	476.5	0.996	0.996	51.15	42.69
*SEM*		71.2	60.1	0.73	0.49	0.10	0.04	73.9	59.0	70.5	56.2	0.001	0.000	18.82	7.55
Main effect means															
CON		303.38	383.75	3.743	4.492	0.774	0.832	382.59	446.91	386.40	449.27	0.996	0.997	42.040	43.373
OAW		283.00	472.42	3.390	5.160	0.749	0.896	345.37	537.79	363.84	539.99	0.996	0.996	35.000	52.604
OAF		328.55	368.18	3.950	4.500	0.757	0.867	383.15	446.91	384.29	448.10	0.996	0.996	51.750	42.204
C.C	−	296.20	416.00	3.728	5.014	0.755	0.901^a^	355.50	474.58	368.00	474.74	0.990	0.997	42.360	45.722
	+	316.30	402.12	3.728	4.415	0.763	0.827^b^	385.50	476.14	388.30	485.58	0.990	0.996	44.300	46.594
P-value	Diet	0.814	0.191	0.729	0.243	0.923	0.243	0.877	0.193	0.962	0.192	0.261	0.400	0.638	0.333
	C.C	0.830	0.755	0.938	0.024	0.815	0.024	0.716	0.984	0.812	0.824	0.866	0.063	0.980	0.867
	Diet × C.C	0.809	0.879	0.937	0.123	0.702	0.123	0.580	0.659	0.649	0.523	0.146	0.071	0.855	0.973

^1^*CON* Control diet, *OAW* Organic acid in water, *OAF* Organic acid in feed, *CONC* Control diet with Coccidial challenge, *OAWC* Organic acid in water with Coccidial challenge; and *OAFC* Organic acid in feed with Coccidial challenge

^a−b^ Mean within each column with no common superscript differ significantly (P < 0.05)

## Discussion

During warm and humid weather, the broiler chickens are mainly affected by the intestinal disease, i.e., Coccidiosis. A low dose of *Eimeria* is present in live vaccines that initiate cellular responses against the parasite after inoculation (Dalloul and Lillehoj 2006). In addition to the energetic cost of immune activation, recycling of the parasite in the intestine creates damage and inflammation predisposing the animal to secondary bacterial infection (Moore 2016). In the present study, coccidial vaccines were administered by the mean of oral gavage that induced the intestinal challenge, and investigated the potential efficacy of OAs blends, inclusion in feed or water, to mitigate the negative impacts of coccidial challenge in broiler chickens raised without antibiotics and anticoccidial drugs. The present study demonstrated that the OAs treatments enhanced the performance by improving intestinal health and immune response against coccidial challenge.

To develop appropriate approaches that help chickens reach maximum growth, it is important to gain more knowledge about the underline mechanisms involved in the integrity and functionality of the intestine (Celi et al. 2017). *Eimeria spp.* the challenge is well known to harm intestinal mucosa, decrease nutrients digestion, and absorption (Shirley and Lillehoj 2012). These microorganisms usually cause inflammation inside the intestine (Chapman 2014), which ultimately reduces feed consumption, rise energy demands (Kogut and Klasing 2009), and result in poor FCR (Dahiya et al. 2006; Immerseel et al. 2009). Certainly, in the present study, the intestinal challenge model was efficient in reducing growth performance, intestinal health, and immune response. In the overall period, BWG and FCR were improved by the inclusion of OAs in feed or water in both challenged and non-challenged groups. Similar results were reported by Stefanello et al. (2020) and Abdelli et al. (2020), who demonstrated that a blend of OAs improved the BWG and FCR in the intestinal challenged group. Furthermore, under the *Eimeria* challenge, a blend of benzoic acid and essential oil enhances the growth performance in broilers (Aristimunha et al. 2016). Better performance could be attributed to the presence of OAs in water and feed, which enhances endogenous GIT enzyme secretion that ultimately produces a positive impact on GIT passage rate and nutrient digestibility in broilers (Khan and Iqbal 2016). Moreover, Coccidal challenged group reduced BW, BWG, FI, and FCR as compared with the non-challenged group. These results are also in agreement with Bortoluzzi et al. (2019) and Belote et al. (2019), who reported poor performance in the coccidial challenged group as compared with the non-challenged group. Therefore, OAs supplementation could be a suitable alternative for anticoccidial or antibiotics due to their ability to improve intestinal health, damaged by coccidial challenge, that ultimately enhances the performance in broilers.

No interaction was found between OAs supplementation and coccidial challenge on plasma immunity indices except IgG and IL-10 were found higher in OAs supplementation in feed with and without coccidial challenge groups, respectively. Similarly, Emami et al. (2013) reported that broilers fed on a diet containing phytase + OAs showed higher IgG. Therefore, it indicates the positive impact of OAs supplementation on IgG. However, the coccidial challenge alone increases all immunoglobulins. Immunoglobulins, i.e., IgM, IgA, and IgG, play a key role in the foreign antigen binding process, and they can cause clumping (agglutination) when they are present on the surface of parasites or microbes, and among them, IgM and IgG cause activation of the complement system (Tizard 1998). The OAs supplementation alone showed no significant effect on serum immune response except IFN-γ was found higher in the broilers supplemented with OAs in the feed, which indicated that the inclusion of OAs in the feed had a positive effect on the immune system with and without coccidial challenge. During avian coccidiosis, regarding cytokine activities, IFN-γ plays a predominant role in bird protection (Yun et al. 2000). In another study, the OAs blend up-regulated the caecal tonsil IFN-γ and ileal IL-6 and IL-10 at d 22 of broiler (Rodríguez-Lecompte et al. 2012). These results indicated the immune-protective effects of OAs in broilers against a coccidial challenge as reported earlier (Abdullahi et al. 2020).

Lesions induced by *Eimeria* species in different parts of birds’ GIT are completely dependent on the infection magnitude and can cause pathological conditions i.e., mild to severe intestinal lesions (TYZZER et al. 1932). It is well known that *E. acervulina* mainly attaches to the duodenum and can extend to the middle part of GIT, *E. maxima* mainly develop in the middle portion and can extend to the lower part of the intestine, and *E. tenella* mainly develops in the last part of GIT, i.e., ceca (Joyner 1978). In the present study, lesions were observed on a jejunal portion of the small intestine, and no jejunal lesions were present in non-challenged groups. In contrast, the challenged control group has shown a higher lesion score, which indicated that the inclusion of OAs in water or feed decreased the jejunal lesion score and improve intestinal health. A similar result was reported by Ali et al. (2014), who showed that the inclusion of butyric acid glycerides in feed decreased the intestinal lesion score. This indicated the anticoccidial effect of OA supplementation, which was already reported by Abbas et al. (2011), who showed the effect of acetic acid against *E. tenella* in broiler chickens. Acetic acid lowers the cecal pH and decreases the impact of oocysts that ultimately reduce intestinal lesions. In the present study, the challenged control group has shown poor growth performance and lower plasma immune response due to intestinal damage and inflammatory process; therefore, a higher number of intestinal lesions were found in the challenged control group (Stefanello et al. 2020).

Intestinal morphology, including VH, CD, and the VH/CD ratio, is an important indicator of intestinal health, recovery, and functionality. It plays a significant role in nutrient digestion and absorption (Celi et al. 2017). Interestingly, jejunal morphology was found similar by the inclusion of OAs alone and their interaction with coccidial challenged. The coccidial challenged group increases CD and decreases the VH/CD ratio, which indicates that coccidial challenge can damage intestinal mucosa (Fernando and McCraw 1973; Oikeh et al. 2019) because decreased VH:CD ratio demonstrating a reduction of the intestinal absorptive capacity and increase of metabolic cost of intestinal epithelium turnover (Xue et al. 2018). Similar findings have been reported regarding the negative effect of coccidial infection on intestinal morphology (Alfaro et al. 2007; Luquetti et al. 2016). However, OAs supplementation decreases CD and increases the VH/CD ratio that indicated the positive effect of OAs on intestinal health. Similar results were reported by Mohammadagheri et al., (2016) in broilers fed on OAs and phytase. As increases in VH and decreases in CD result in a high VH/CD ratio, which is an indicator that the broiler chickens have mature enterocytes at the villus tips, a balanced enterocyte migration, and sloughing because VH/CD ratio can be directly correlated with the balance of VH and CD. Moreover, coccidiosis causes a lower number of GC that indicated mucosal atrophy and epithelial cell necrosis due to coccidiosis. The GCs are a major source of mucins that played a role as the first line of defense by maintaining the intestinal barrier (Golder et al. 2011). OAs as dietary supplements and water acidifiers resulted in a higher number of jejunal goblet cells, which lead to the stimulation and production of the mucus layer (Strous and Dekker 1992).

Besides intestinal morphological parameters, TJP also played an important role in the passage of antigens and pathogens through the intestinal epithelium (Broom 2018). In jejunum and cecal tonsil, TJP gene expression showed no significant interaction between OAs supplementation and the coccidial challenge. Although OAs supplementation with and without coccidial challenge also had no significant effect on TJP gene expression, OAs supplementation showed numerically higher TJP expression. Similarly, in under-challenged conditions, the upregulation of CDLN1 and OCLN in broilers supplemented with OA + EO was previously reported by Stefanello et al., (2020). On the other hand, without coccidial challenged, different scientists have reported the positive effect of OAs supplementation on intestinal TJP gene expression (Corfield et al. 2000; Pham et al. 2020; Dai et al. 2021). Mcknight et al., (2019) reported a blend of fatty acids, OAs, and phytochemicals supplementation upregulated CDLN1 and ZO-1 in broilers. ZO-1 anchors CLDN and OCLN to intracellular actin, facilitating crosslinks between the actin cytoskeletons and the transmembrane proteins (Förster 2008). Therefore, upregulation of these TJP enhances epithelial tightness and improves intestinal permeability (Pérez-Bosque et al. 2006) that authenticates OAs supplementation that could control the intestinal permeability.

The GIT is tightly harbored by microbes in poultry, having to be in intensive and close interaction with the host and food particles. They are mainly involved in the nutrients exchange and modify the bird’s intestinal immunity, physiology, and morphology (Yadav and Jha 2019). SCFAs are produced by microorganisms in the GIT of the bird. These SCFAs are involved in mucin production, intestinal immune response, and intestinal blood flow regulation, and stimulate the proliferation and growth of enterocytes (Iacob et al. 2019). Few studies have been reported regarding the effect of *Eimeria* infection on microbial community richness (Stanley et al. 2012; Zhou et al. 2017; Macdonald et al. 2017). These studies reported that intestinal challenge itself does not have a major effect on richness. The model used in the present study has not been investigated previously. Though phylum, genus, and species level were found similar among all the experimental groups, coccidial challenge reduces the *Lactobacillus reuteri* on 18 d. However, OAs supplementation in water and feed increases the abundance of *Lactobacillus reuteri* in the non-challenged group. As *Lactobacillus reuteri* isolated from the digestive tract of broiler chickens can act as probiotics (Jha et al. 2020) and had antimicrobial activity resulting in reduced colonization of *Campylobacter jejuni* in the ileum of broilers (Ghareeb et al. 2012). Therefore, *Lactobacillus reuteri* enhances growth performance, intestinal health, immunity level, and GIT histomorphology (Nakphaichit et al. 2011; Ahmed et al. 2014). Similarly, during the grower phase, *Cyanobacteria* (phylum level), *Cyanobacteria, Lactobacillus,* and *unidentified Cyanobacteria* (Genus Level) were found higher in the non-challenged as compared to the coccidial challenge group. Similar results were reported by Bortoluzz et al., (2018) as microbiota could be disrupted in the challenged broilers. Alpha diversity was also found similar among all experimental groups during both phases except the Simpson was found higher in the non-challenged group than the coccidial challenge group. Moreover, alpha and beta diversity was also found alike between OAs supplementation groups and coccidial challenge groups. Similar results have been reported as the alpha- (Pham et al. 2020; Abdelli et al. 2020) and beta- (Abdelli et al. 2020) diversity was found similar by the inclusion of OAs and EOs in broiler challenged with necrotic enteritis. On the other hand, the coccidial challenge had a pronounced effect on the beta diversity on 29 d. Latorre et al., (2018) reported similar results as coccidia and NE can produce a significant effect on the beta diversity in broilers.

Conclusively, the coccidial challenge model was found effective in causing a disturbance in the homeostasis of the intestine that mainly affects growth performance, plasma immune indices, and intestinal health of broiler chickens. Coccidial challenge negatively affects ileal microbiota, however, no significant effect was found with OAs supplementation with and without coccidial challenge. However, the protected blend of organic acids as water or feed additive showed improved or similar responses to control in neutralizing the negative effects caused by the coccidial challenge. Overall, in this experiment, two different commercial blends of OAs were used by two different inclusion methods, i.e., water and feed. Both OAs blends have shown similar results on birds’ health with and without the coccidial challenge. The efficacy of OAs blends majorly depends on the solubility and acid-binding capacity (ABC) of water (Coban 2020) and feed ingredients (Mohammadpour et al. 2014), respectively. Therefore, positive results of current OAs blends might be due to their high solubility in water and optimum ABC of feed ingredients. Similar results were reported by various scientists who were used similar OAs blends as water acidifiers (Hu et al. 2020) and feed additives (Hogan and Page 2017). On the other hand, a feasible synergistic effect of the OAs blend present in water acidifier or feed additive might be a reason for better birds’ performance (Wang et al. 2019). However, it is hard to point out the exact source of greater performance in broilers with and without coccidial challenge. It might be due to the inclusion method or OAs blend present in water acidifier or feed additive. Further study is needed to investigate the precise cause of the positive response of OAs by using similar OAs blends as water acidifiers and feed additives.

## Data Availability

The datasets can be found in NCBI Sequence Read Archive database (Accession No. PRJNA745096).
